# Genetic association tests in family samples for multi-category phenotypes

**DOI:** 10.1186/s12864-021-08107-x

**Published:** 2021-12-04

**Authors:** Shuai Wang, James B. Meigs, Josée Dupuis

**Affiliations:** 1 Pfizer Inc, Global Product Development, Groton, CT 06340 USA; 2 Division of General Internal Medicine, Massachusetts General Hospital, Boston, MA 02114 USA; 3 Department of Medicine, Harvard Medical School, Boston, MA 02115 USA; 4 Programs in Metabolism and Medical & Population Genetics, Broad Institute of MIT and Harvard, Cambridge, MA 02142 USA; 5 Department of Biostatistics, Boston University School of Public Health, Boston, MA 02118 USA

**Keywords:** EGEE, Score test, Wald test, Framingham heart study, Family samples, Categorical, Multinomial, Ordinal, GWAS, Sequencing

## Abstract

**Background:**

Advancements in statistical methods and sequencing technology have led to numerous novel discoveries in human genetics in the past two decades. Among phenotypes of interest, most attention has been given to studying genetic associations with continuous or binary traits. Efficient statistical methods have been proposed and are available for both types of traits under different study designs. However, for multinomial categorical traits in related samples, there is a lack of efficient statistical methods and software.

**Results:**

We propose an efficient score test to analyze a multinomial trait in family samples, in the context of genome-wide association/sequencing studies. An alternative Wald statistic is also proposed. We also extend the methodology to be applicable to ordinal traits. We performed extensive simulation studies to evaluate the type-I error of the score test, Wald test compared to the multinomial logistic regression for unrelated samples, under different allele frequency and study designs. We also evaluate the power of these methods. Results show that both the score and Wald tests have a well-controlled type-I error rate, but the multinomial logistic regression has an inflated type-I error rate when applied to family samples. We illustrated the application of the score test with an application to the Framingham Heart Study to uncover genetic variants associated with diabesity, a multi-category phenotype.

**Conclusion:**

Both proposed tests have correct type-I error rate and similar power. However, because the Wald statistics rely on computer-intensive estimation, it is less efficient than the score test in terms of applications to large-scale genetic association studies. We provide computer implementation for both multinomial and ordinal traits.

**Supplementary Information:**

The online version contains supplementary material available at 10.1186/s12864-021-08107-x.

## Background

Genetic association tests for continuous or binary phenotypes have uncovered many susceptibility genes or variants related to diseases. Various methods and efficient software have been developed and used for continuous and binary traits. For family samples, due to the correlation between relatives and violation of the independence assumption of ordinary linear regression, some alternative approaches were proposed. For example, Therneau and colleagues developed an R package (coxme) implementing linear mixed effects model to evaluate the association between a genetic variant and a continuous trait or survival outcome accounting for correlation present in family samples. Similar extensions to account for familial correlation using mixed effects models have been proposed for gene-based association tests [1]. The progress in family sample designs has been restricted mostly to quantitative traits or binary traits. However, methods are needed to study categorical traits with more than two categories in family samples. For example, the phenotype diabesity has been defined as a four-category (diabetes & obesity, diabetes but no obesity, obesity but no diabetes and no diabetes & no obesity) variable constructed jointly from type 2 diabetes and obesity. Currently, approaches for genetic association analysis of such multinomial traits are limited. Zhang and colleagues [2] proposed a proportional odds logistic model which allows for the inclusion of covariates. However, it has a few limitations. First, this approach is restricted to nuclear families and cannot handle complex family structures. Second, no software implementation has been made publicly available. Diao and Lin [3] proposed a general framework for linkage and association tests for ordinal traits. Their method utilized adaptive Gaussian quadrature to approximate the maximum log-likelihood and a likelihood ratio test was proposed to test the hypothesis of no association between a genetic variant and an ordinal trait of interest. Again, this approach also has not been widely used due to lack of computer-efficient software and the fact that the likelihood ratio test is computationally intensive. Another possible option is to use the SAS generalized linear mixed models (GLMM) procedure, which can incorporate a kinship matrix. However, in real applications, the current implementation of the GLMM cannot handle extended families due to the computational burden. More recently, Wang and colleagues [4] proposed a Bayesian framework incorporating kinship matrix as a random effect, which however can not be applied to large-scale genetic study because of lack of computational efficiency. Bi and colleagues [5] proposed a computer-efficient framework (POLMM), specifically for ordinal traits. Because it doesn’t allow for a user-provided kinship matrix, such as the one estimated from pedigree or using a typical genetic software, this will be a limitation for family-based cohort studies with known relationships. Our proposed method is complementary to these two approaches as it can be applied to family samples without available genome-wide data to compute a GRM, and without the proportional odds assumption. In this paper, we propose a computationally efficient score test based on extended generalized estimating equations (EGEE) for large-scale genetics studies of multi-category phenotypes accounting for familial correlation. We evaluate our approach using simulations and apply it to a genome-wide scan to identify genetic variants associated with diabesity, a four-category phenotype, with the healthy referent category being no diabetes and no obesity and the unhealthiest category, “diabese” (diabetes and obesity), having a prevalence of at least 25% in several countries [6].

## Results

### Type-I error

The results of family-based and unrelated samples are summarized in Table 1-2 respectively. Both the score and Wald tests have well-controlled type-I error rates across all MAF scenarios except for rare variants. This conclusion applies to both family-based and unrelated designs. The multinomial logistic regression, which ignores familial correlation, returns an inflated type-I error rate in the presence of related individuals, although its type-I error rate for unrelated study design is well-controlled. In the application to ordinal trait (Table 3), robust score test preserves the type-I error in all MAF scenarios although the simulated phenotype distribution is highly unbalanced. The Wald test is only very slightly inflated for very rare variants when evaluated at 0.0001. We have also generated QQ-plots (Additional File 3) for the robust score test and the simplified score test for results from all MAF scenarios for both multinomial and ordinal traits when applied to family-based samples. The QQ-plots are consistent with the empirical type-I error summarized in the tables below.

**Table 1:** Simulation results of type-I error for family-based samples

MAF	Robust Score test			Wald test			Logistic regression (LRT)		
	α = 0.01	α = 0.001	α = 0.0001	α = 0.01	α = 0.001	α = 0.0001	α = 0.01	α = 0.001	α = 0.0001
0.01	0.014	0.0020	0.0003	0.012	0.0024	0.00058	0.023	0.0023	0.0006
0.02	0.013	0.0020	0.0003	0.010	0.0011	0.0003	0.021	0.0027	0.0004
0.03	0.012	0.0017	0.0002	0.009	0.0012	0.0002	0.022	0.0025	0.0004
0.04	0.011	0.0014	0.0002	0.007	0.0010	0.0002	0.022	0.0025	0.0006
0.05	0.011	0.0013	0.0002	0.011	0.0008	0.0002	0.021	0.0026	0.0002
0.1	0.011	0.0010	0.0001	0.009	0.0008	0.0001	0.021	0.0024	0.0003
0.2	0.010	0.0010	0.0001	0.010	0.0010	0.0001	0.019	0.0033	0.0004
0.3	0.010	0.0010	0.0001	0.011	0.0013	0.0001	0.021	0.0033	0.0011

**Table 2:** Simulation results of type-I error for unrelated samples

MAF	Score test		Wald test		Logistic regression (LRT)	
	α = 0.01	α = 0.001	α = 0.01	α = 0.001	α = 0.01	α = 0.001
0.01	0.011	0.0010	0.008	0.0006	0.011	0.0011
0.02	0.010	0.0016	0.010	0.0016	0.012	0.0014
0.03	0.012	0.0012	0.011	0.0010	0.011	0.0014
0.04	0.011	0.0010	0.010	0.0010	0.011	0.0010
0.05	0.010	0.0010	0.006	0.0004	0.010	0.0005
0.1	0.010	0.0010	0.010	0.0008	0.010	0.0010
0.2	0.010	0.0010	0.009	0.0004	0.010	0.0010
0.3	0.009	0.0011	0.009	0.0006	0.010	0.0010

**Table 3:** Simulation results of type-I error for family-based samples for ordinal traits

MAF	Robust Score test			Wald test		
	α = 0.01	α = 0.001	α = 0.0001	α = 0.01	α = 0.001	α = 0.0001
0.01	0.010	0.0008	0.00009	0.012	0.0013	0.00019
0.02	0.009	0.0008	0.00008	0.011	0.0011	0.00013
0.03	0.010	0.0010	0.00009	0.011	0.0012	0.00012
0.04	0.009	0.0009	0.00008	0.010	0.0011	0.00011
0.05	0.010	0.0009	0.00010	0.011	0.0011	0.00014
0.1	0.010	0.0010	0.00009	0.010	0.0011	0.00009
0.2	0.010	0.0009	0.00009	0.010	0.0010	0.00012
0.3	0.009	0.0009	0.00010	0.010	0.0010	0.00012

### Power evaluation

The results of family-based and unrelated samples are summarized in Tables 4 and 5**,** respectively. Because we have concluded that multinomial logistic regression leads to inflated type-I error rates, the power rate of multinomial logistic regression is not evaluated for family-based samples (Table 4). The score and Wald tests have approximately the same power rate for each scenario (MAF, study design). The logistic regression using LRT has approximately the same power as the other two approaches in unrelated samples.

**Table 4:** Power results for family-based samples

MAF	α=0.01		α=0.001		α = 5 × 10^−8^	
0.01	score	97.2	score	92.4	score	42.5
	Wald	96.7	Wald	90.2	Wald	29.8
0.02	score	96.5	score	89.1	score	33.0
	Wald	96.6	Wald	86.4	Wald	24.4
0.03	score	95.5	score	87.5	score	25.6
	Wald	95.1	Wald	84.6	Wald	20.5
0.04	score	94.9	score	85.4	score	23.4
	Wald	94.6	Wald	82.4	Wald	17.7
0.05	score	94.3	score	83.6	Score	20.9
	Wald	93.5	Wald	81.2	Wald	15.8
0.1	score	93.0	score	78.6	score	13.8
	Wald	94.3	Wald	79.6	Wald	11.6
0.2	score	89.4	score	71.7	score	7.6
	Wald	91.1	Wald	74.3	Wald	8.0
0.3	score	87.4	score	68.2	score	6.4
	Wald	89.0	Wald	71.0	Wald	6.5

**Table 5:** Power results for unrelated samples

MAF	α=0.01		α=0.001		α = 5 × 10^−8^	
0.01	score	95.0	score	85.8	score	26.5
	Wald	94.1	Wald	82.8	Wald	15.6
	Logistic (LRT)	92.9	Logistic (LRT)	79.6	Logistic (LRT)	11.4
0.02	score	93.3	score	82.3	score	20.0
	Wald	92.8	Wald	80.6	Wald	14.6
	Logistic (LRT)	91.6	Logistic (LRT)	77.4	Logistic (LRT)	10.6
0.03	score	92.7	score	81.1	score	15.9
	Wald	92.4	Wald	79.5	Wald	12.7
	Logistic (LRT)	91.2	Logistic (LRT)	76.8	Logistic (LRT)	9.5
0.04	score	92.4	score	79.2	score	14.1
	Wald	92.0	Wald	78.2	Wald	11.3
	Logistic (LRT)	90.8	Logistic (LRT)	75.4	Logistic (LRT)	8.6
0.05	score	92.0	score	77.9	score	13.1
	Wald	91.9	Wald	77.3	Wald	10.9
	Logistic (LRT)	90.9	Logistic (LRT)	74.7	Logistic (LRT)	8.2
0.1	score	91.3	score	75.7	score	10.4
	Wald	91.0	Wald	74.9	Wald	9.5
	Logistic (LRT)	90.3	Logistic (LRT)	73.2	Logistic (LRT)	7.8
0.2	score	89.9	score	73.2	score	8.1
	Wald	89.7	Wald	73.0	Wald	7.5
	Logistic (LRT)	89.3	Logistic (LRT)	71.9	Logistic (LRT)	6.8
0.3	score	89.2	score	72.2	score	7.0
	Wald	89.3	Wald	71.8	Wald	6.5
	Logistic (LRT)	89.0	Logistic (LRT)	71.5	Logistic (LRT)	6.3

### Data analysis

**Fig. 1:**
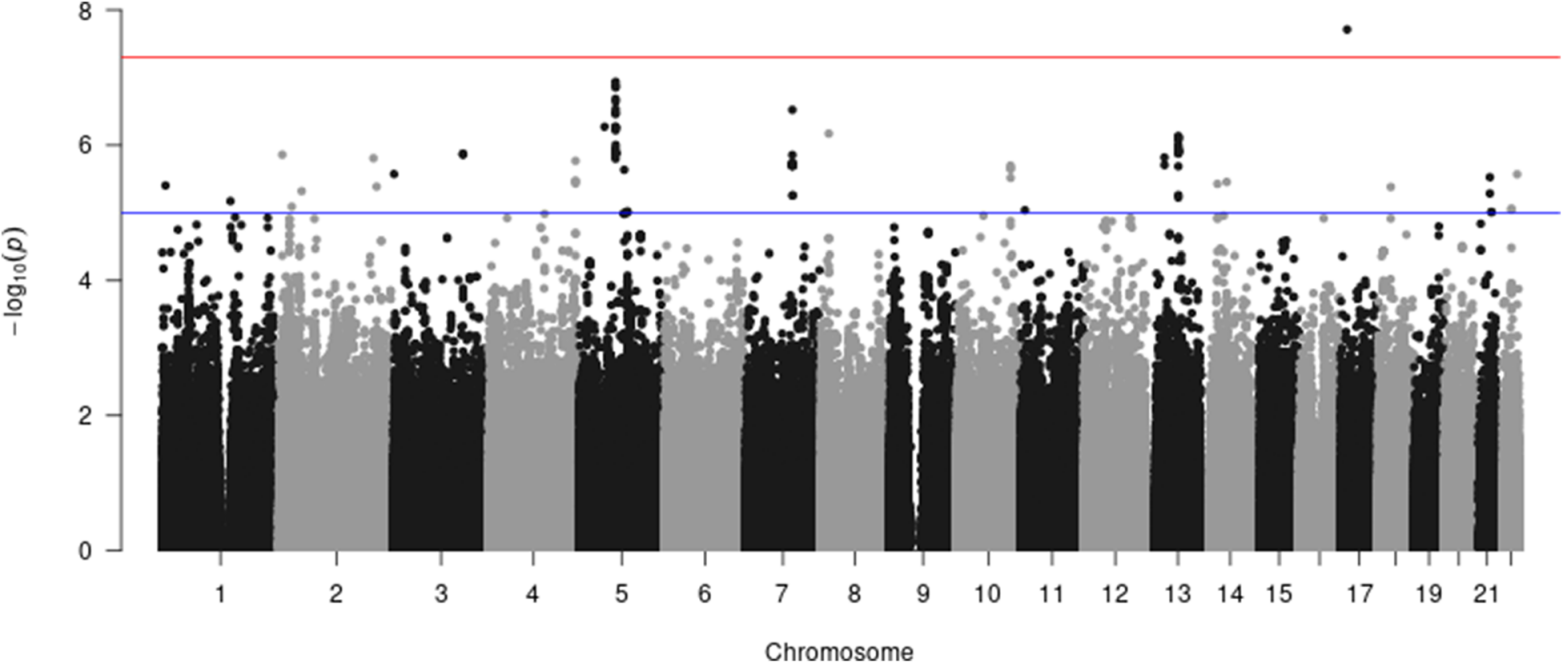
Manhattan plot of diabesity using the FHS data and Hapmap imputed genotypes

**Fig. 2:**
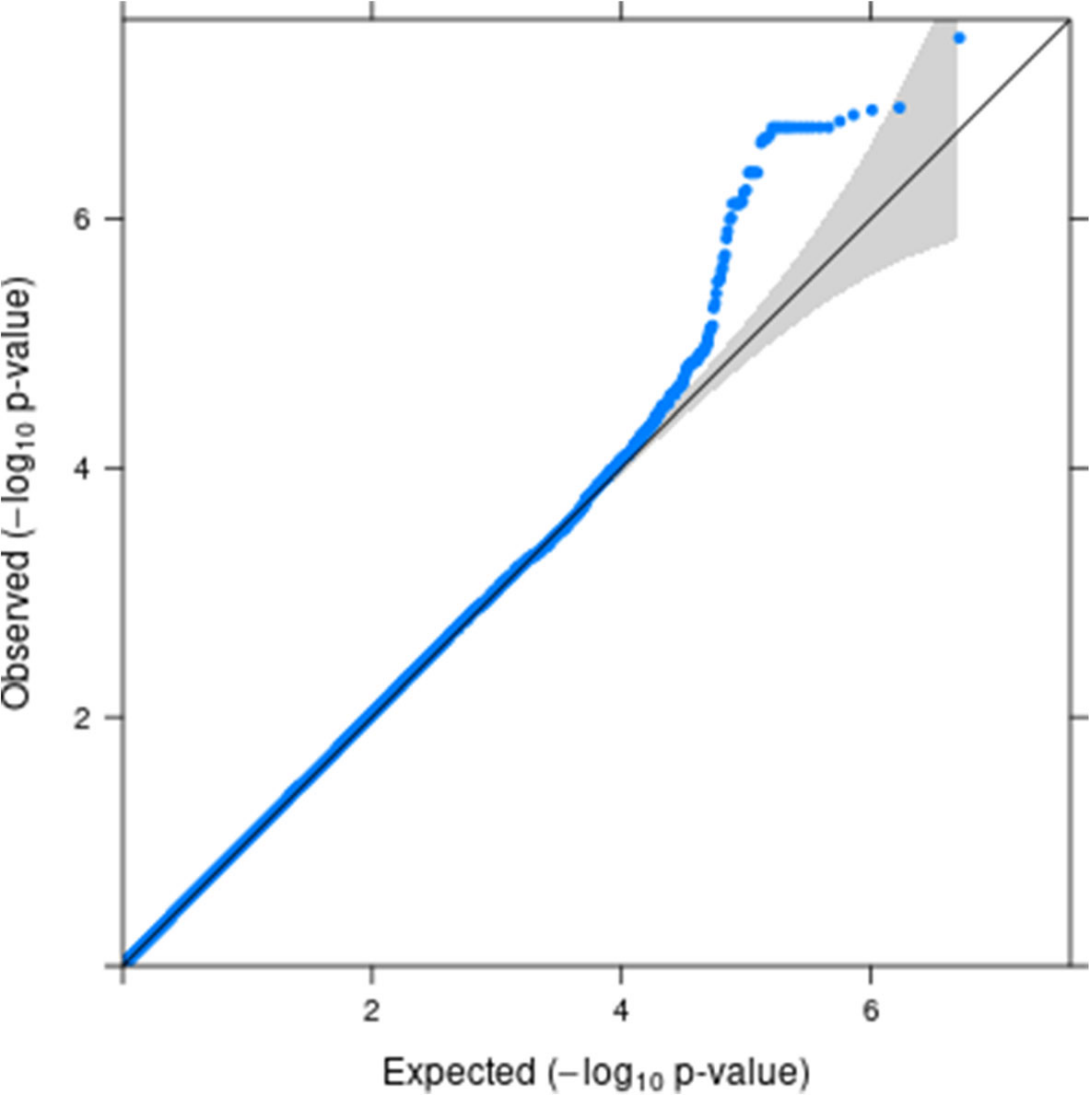
QQ-plot of diabesity using the FHS data and Hapmap imputed genotypes

Low-frequency (MAF < 0.01) and poorly imputed variants (imputation ratio < 0.3) have been excluded to avoid spurious results. All results are presented in the Manhattan plot (Fig. 1**.,** and Manhattan plots for diabetes, obesity in Additional File 3) and QQ-plot (Fig. 2**.**). The variants that have reached a genome-wide significance threshold of 5 × 10^−8^ or a suggestive threshold of 4 × 10^−7^ (calculated as 1/number of tests = 1/2542166) are summarized in Table 6. All variants in Table 6 are located within the ***CYP3A43***, ***AP3B1*** and ***LOC105370246*** genes. ***AP3B1*** is known to have variants associated with fasting insulin and HOMA-IR in African Americans without diabetes [7]. The direct association between ***LOC105370246*** and dibestes or obesity is not known in literature. ***CYP3A43*** gene encodes a member of the cytochrome P450 superfamily of liver enzymes. Although the direct relationship between ***CYP3A43*** and diabetes/obesity was not well known, some variants located in ***CYP3A4*** have been identified in previous studies to be associated with relevant metabolism traits. For instance, one study in 2011 [8] indicated diabetes is associated with a significant decrease in hepatic ***CYP3A4*** enzymatic activity and protein level. Several studies have demonstrated nonalcoholic fatty liver disease and diabetes are associated with decreased expression of the protein encoded by this gene in human livers [9, 10]. Two variants on ***CYP3A43*** were identified to be associated with Ticagrelor levels in individuals with acute coronary syndromes treated with ticagrelor [11] and serum metabolite measurement [12] respectively. Because this gene might have clinical value for treating chronic metabolic diseases such as nonalcoholic fatty liver disease [13], future research efforts targeting this gene area are worthwhile. Additional information about this region might be discovered with targeted sequencing.

**Table 6:** Top SNPs and the closest genes

Chr	Lead SNP	***p***-value	bp (GRCh38)	Loci (closest gene)
5	rs16875172	1.17*10^(− 7) to 5.34*10^(− 7)	5:77422013–5:77559950	***AP3B1***
7	rs528144	2.99*10^(− 7) to 5.56*10^(− 6)	7:99257162–7:99918674	***CYP3A43***
13	rs1925751	7.34*10^(−7) to 5.97*10^(− 6)	13:66763957–13: 66795551	***LOC105370246***

For target validation purpose, we have performed two additional GWAS of diabetes and obesity respectively using our approach (Manhattan plots in Additional File 3). The plots confirm that all signals are observed from the combined phenotype and not driven by a single binary trait (diabetes or obesity).

**Fig. 3:**
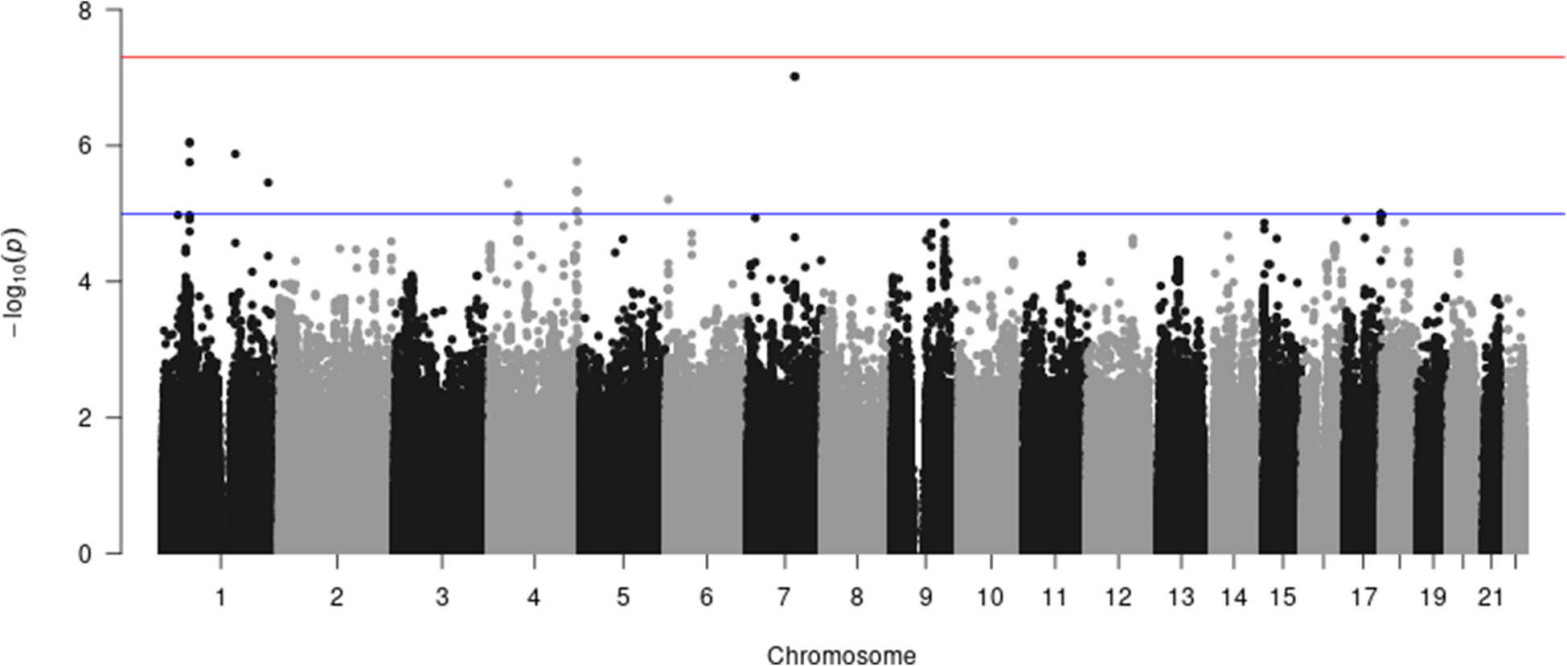
Manhattan plot of “ordinal” diabesity using the FHS data and Hapmap imputed genotypes (proposed ordinal approach)

We apply our ordinal approach to the secondary outcome (“ordinal” diabesity) and compare to results obtained from POLMM, an approach for ordinal trait. We observe that the results are similar with small differences (Fig. 3. and 4). Compared to results obtained from the multinomial trait (Fig. 1**.**), the ordinal trait highlights one region near ***DAB1*** on Chromosome 1 and one region near ***LOC107986327*** on Chromosome 4 of potential interest in the search for genes associated with “ordinal” diabesity.

**Fig. 4:**
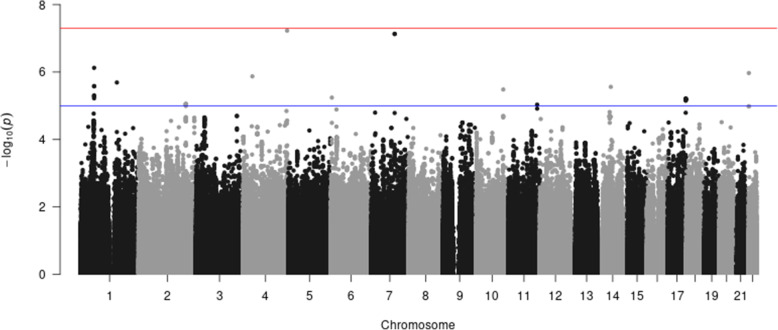
Manhattan plot of “ordinal” diabesity using the FHS data and Hapmap imputed genotypes (POLMM approach)

## Discussion

The proposed score test offers advantages over the Wald test and the multinomial logistic regression in the following aspects. First, it is more computationally efficient, especially for large-scale genetic studies such as GWAS, or sequencing studies because the iterative Fisher’s scoring algorithm is only applied once under the null hypothesis while the iterative algorithm is implemented for each variant when computing the Wald test statistic. Therefore, for a large-scale genetic study, the Wald test will be less computationally efficient than the score test. We have summarized the computing time for the score and Wald tests in Table 7 for different sample sizes as implemented in R functions using a 3-category multinomial phenotype on a i7-8565u processor with 16GB RAM. Second, the simulation studies show that the type-I error of both the score and Wald tests is well controlled for most scenarios. In contrast, the multinomial logistic regression results in a very inflated type-I error rate for family-based design when the familial correlation is ignored, and therefore it is not recommended for family-based studies. When the phenotypes are extremely unbalanced, e.g. the allocation ratio of the 4 categories is approximately 2.5:1:10:23, both score and Wald tests can result in slightly inflated type-I error for rare variants in the simulation studies. This conclusion has been noted in most approaches [5]. However, when the phenotype distribution is more balanced, the tests return valid type-I error rates for all MAF scenarios, as demonstrated in the QQ-plot of the FHS data analysis (Fig. 2**.**). We have observed that the type-I error of ordinal traits is very robust to an unbalanced distribution of the phenotype for all MAF scenarios (Table 3), as indicated by the calculated type-I error rate obtained from 500,000 simulations by treating the simulated phenotype as an ordinal trait. Lastly, the score test has approximately the same power as the Wald test, under the scenarios we evaluated.

**Table 7:** Computing time of robust score and Wald tests on a i7-8565u processor with 16GB RAM

Sample size	Robust score test	Wald test
5000 (182 families)	3.09 s (initial) + 1.17 s per SNP	3.44 s per SNP
10,000 (364 families)	4.47 (initial) + 2.45 s per SNP	6.92 s per SNP
20,000 (728 families)	5.47 (initial) + 5.25 s per SNP	10.95 s per SNP

It is worth noting that the EGEE are simply reduced to the score equations of generalized linear models for a multinomial variable when applied to unrelated samples. Because the same iteratively reweighted least square method is employed under this particular circumstance, the parameter estimates are identical to those obtained using a generalized linear model function for multinomial variables. This equivalence enhances the applicability of this approach to a general population, regardless of the underlying study design.

The score test can be readily extended to ordinal traits (i.e. categorical traits for which the values are ordered.) in family samples. Due to the nature of the ordinal regression model, fewer regression parameters are estimated. Because applications to ordinal traits are a special case of the general framework proposed with reduced complexity, the validity of simulation results should hold when applied to ordinal traits. When K = 2, i.e. an ordinal trait with only two categories, the estimates will be the same when using either multinomial or ordinal function, i.e. estimates of a binary logistic regression accounting for familial correlation.

Our proposed approaches have enabled the identification of a few loci associated with diabesity. As discussed, none of the signals were driven solely by one of the two binary traits (diabetes or obesity). Targeted sequencing might reveal more information, by providing a more comprehensive overview of rare and low-frequency variants in that specific regions. We also provide a comparison of our ordinal approach to POLMM for an ordinal trait and found that both approaches have revealed similar regions of association.

## Conclusions

Score tests should be considered for large-scale genetic association testing due to their computational advantage. Because the Wald test also has valid type-I error rates and its computational efficiency is comparable to the score test (Table 7), if computing resources allow, the Wald test can also be applied for large-scale genetic studies. As illustrated using Framingham heart study data, the proposed score test has enabled the identification of several loci associated with diabesity. One of the drawbacks of the score test is the lack of effect estimates. When only a handful of associated variants are identified from a genetic association study, the effect size and statistical significance of each variant can be estimated using the Wald test. In addition to the multinomial application, we have also provided a computer implementation for ordinal traits in Additional File 2. Although we presented association results from additively coded genetic variants, the application and implementation are not restricted to SNPs, but also applicable to a genetic risk score, weighted-sum gene test [14], and other genetic summary measures.

## Methods

Assuming that there are N independent families (*i* = 1, …, N), with *n*_*i*_ individuals in family *i* and a total of $n={\sum}_{i=1}^N{n}_i$ subjects, the basic model for a K-category (multinomial) trait, with the Kth level chosen as the reference level, is written as,

$$ g\left({Y}_{ij}=\left.k\right|{\boldsymbol{X}}_{ij},{G}_{ij}\right)={\alpha}_k+{\beta}_k{G}_{ij}+{\boldsymbol{X}}_{ij}^T{\boldsymbol{\gamma}}_{\boldsymbol{k}}\kern4em \mathrm{k}=1,\dots, \left(\mathrm{K}\hbox{-} 1\right) $$

The n × 1 response variable **Y** has K unordered levels, i.e. k = 1, …, K, resulting in (K-1) equations; ***G*** is the genotype vector of size n × 1; **X** is the n × q covariates matrix; **α** = (*α*_1_, …, *α*_*k*_, …, *α*_*K* − 1_)^*T*^ is the intercept vector for the (K-1) equations; **β** = (*β*_1_, …, *β*_*k*_, …, *β*_*K* − 1_)^*T*^ is the effect size vector of the genotype in the (K-1) equations; and **γ** = (***γ***_**1**_, …, ***γ***_***k***_, …, ***γ***_***K −*** **1**_) are the parameters of the covariates **X**, for the (K-1) equations with a dimension of q × 1 for each ***γ***_***k***_**.** Although there are a variety of choices for the link function g, here we demonstrate with the canonical link function, the general logit, i.e.

$$ g\left({Y}_{ij}=k|{\boldsymbol{X}}_{ij},{G}_{ij}\right)=\log \frac{P\left({Y}_{ij}=k|{\boldsymbol{X}}_{ij},{G}_{ij}\right)}{P\left({Y}_{ij}=K|{\boldsymbol{X}}_{ij},{G}_{ij}\right)}. $$

### Extended generalized estimating equations (EGEE)

We adopt the idea of EGEE previously proposed [15, 16] to approximate the likelihood using quasi-likelihood, to handle correlated observations. The variance of the response variable *Y*_*ij*_, is defined using (K-1) indicator variables as follows: ***z***_*ij*_ = [I(*Y*_*ij*_ = 1), …, I(*Y*_*ij*_ = (*K* − 1))] ’. The expected value of ***z***_*ij*_ is E[***z***_*ij*_] = [P(*Y*_*ij*_ = 1), …, P(*Y*_*ij*_ = (*K* − 1))]′ and the variance of ***z***_*ij*_ can be derived as:

$$ \operatorname{var}\left({\boldsymbol{z}}_{ij}\right)=\left(\begin{array}{ccc}\mathit{\operatorname{var}}\left(I\left({Y}_{ij}=1\right)\right)& \dots & \mathit{\operatorname{cov}}\left(I\left({Y}_{ij}=1\right),I\left({Y}_{ij}=\left(K-1\right)\right)\right)\ \\ {}\vdots & \mathbf{\ddots}& \vdots \\ {}\mathit{\operatorname{cov}}\left(I\Big({Y}_{ij}=\left(K-1\right)\right),I\left({Y}_{ij}=1\right)\Big)\ & \dots & \mathit{\operatorname{var}}\left(I\left({Y}_{ij}=\left(K-1\right)\right)\right)\end{array}\right)=\left(\begin{array}{ccc}\mathrm{P}\left({Y}_{ij}=1\right)\left(1-\mathrm{P}\left({Y}_{ij}=1\right)\right)& \dots & -\mathrm{P}\left({Y}_{ij}=1\right)\mathrm{P}\left({Y}_{ij}=\left(K-1\right)\right)\\ {}\vdots & \mathbf{\ddots}& \vdots \\ {}-\mathrm{P}\left({Y}_{ij}=\left(K-1\right)\right)\mathrm{P}\left({Y}_{ij}=1\right)\ & \dots & \mathrm{P}\left({Y}_{ij}=\left(K-1\right)\right)\left(1-\mathrm{P}\left({Y}_{ij}=\left(K-1\right)\right)\right)\end{array}\right) $$

Let ***R*** = *r****J*** where ***J*** is a matrix of ones with a dimension of (K-1) by (K-1), and *r* is an unknown correlation parameter to be estimated with value between −1 and 1. The implementation of the approach provided in Additional File 1 can also accommodate two-parameter ***R*** with diagonal elements set to *r*1 and all off-diagonal elements set to *r*2. The matrix ***R*** is used to model the correlation between any two individuals in the same family along with the use of relationship matrix, such that ***R***_*i*_ = **Φ**_*i*_ ⨂ ***R*** ( **Φ**_*i*_ is the relationship matrix of the i-th family defined as twice the kinship matrix), similar to how the familial correlation was handled in previous publications [17, 18]. ***V***_***i***_**,** the overall variance matrix of ***z***_*i*._ for the i-th independent family is constructed as *sd*(***z***_*i*._)***R***_*i*_*sd*(***z***_*i*._) with the variance of each subject var(***z***_*ij*_) (*j* = 1, …, *n*_*i*_), as derived above,

where

$$ sd\left({\boldsymbol{z}}_{\boldsymbol{i}.}\right)=\left(\begin{array}{ccc} sd\left({\boldsymbol{z}}_{\boldsymbol{i}\mathbf{1}}\right)& 0& 0\\ {}0& \ddots & 0\\ {}0& 0& sd\left({\boldsymbol{z}}_{\boldsymbol{i}{\boldsymbol{n}}_{\boldsymbol{i}}}\right)\end{array}\right) $$

and

$$ sd\left({\boldsymbol{z}}_{\boldsymbol{ij}}\right)=\left(\begin{array}{ccc}\sqrt{\operatorname{var}\left(I\left({Y}_{ij}=1\right)\right)}& 0& 0\\ {}0& \ddots & 0\\ {}0& 0& \sqrt{\operatorname{var}\left(I\left({Y}_{ij}=K-1\right)\right)}\end{array}\right)\forall j=1,\dots, {n}_i. $$

The following score equations of EGEE [15, 16, 19] are used to estimate the regression parameters ***θ*** = (*α*_1_, *β*_1_, ***γ***_**1**_, …, *α*_*K* − 1_, *β*_*K* − 1_, ***γ***_***K*** **− 1**_) and the correlation parameter *r*.

$$ \boldsymbol{U}={\sum}_{i=1}^N{\boldsymbol{U}}_{\boldsymbol{i}}\left(\boldsymbol{\theta}, r\right)={\sum}_{i=1}^N\left(\begin{array}{cc}{\boldsymbol{D}}_{\boldsymbol{i}}^{\prime }& \mathbf{0}\\ {}\mathbf{0}& {\boldsymbol{F}}_{\boldsymbol{i}}^{\prime}\end{array}\right)\left(\begin{array}{cc}{\boldsymbol{V}}_{\boldsymbol{i}}^{-\mathbf{1}}& \mathbf{0}\\ {}\mathbf{0}& \boldsymbol{I}\end{array}\right)\left(\begin{array}{c}{\boldsymbol{y}}_{\boldsymbol{i}}-{\boldsymbol{\mu}}_{\boldsymbol{i}}\\ {}{\boldsymbol{s}}_{\boldsymbol{i}}-{\boldsymbol{\sigma}}_{\boldsymbol{i}}\end{array}\right)=\mathbf{0} $$

where the *n*_*i*_(*K* − 1) × (2 + *q*)(*K* − 1) matrix ***D***_***i***_ is stacked vertically from ***D***_***ij***_ (j = 1, …, *n*_*i*_) and defined as ${\boldsymbol{D}}_{\boldsymbol{ij}}=\frac{\partial E\left[{z}_{ij}\right]}{\partial {\boldsymbol{\theta}}^{\prime }}={\left(\frac{\partial P\left({Y}_{ij}=1\right)}{\partial \boldsymbol{\theta}},\dots, \frac{\partial P\left({Y}_{ij}=K-1\right)}{\partial \boldsymbol{\theta}}\right)}^{\prime }$; ***F***_***i***_ is the vectorized $\frac{\partial {\boldsymbol{V}}_{\boldsymbol{i}}^{-\mathbf{1}}}{\partial r}$ with a dimension of ${n}_i^2{\left(K-1\right)}^2$ by 1, ***I*** is an identity matrix with a size of ${n}_i^2{\left(K-1\right)}^2$ and ***σ***_***i***_ is the vectorized version of ***V***_***i***_. Similarly, ***s***_***i***_ is vectorized version derived from the following:

$$ \left(\begin{array}{c}{\boldsymbol{e}}_{\boldsymbol{i}\mathbf{1}}\\ {}\boldsymbol{\vdots}\\ {}{\boldsymbol{e}}_{\boldsymbol{i}{\boldsymbol{n}}_{\boldsymbol{i}}}\end{array}\right)\left({\boldsymbol{e}}_{\boldsymbol{i}\mathbf{1}}^{\prime}\kern0.5em \dots \kern0.5em {\boldsymbol{e}}_{\boldsymbol{i}{\boldsymbol{n}}_{\boldsymbol{i}}}^{\prime}\right) $$

where ${\boldsymbol{e}}_{\boldsymbol{ij}}=\left({e}_{ij}^1\kern0.5em \dots \kern0.5em {e}_{ij}^{K-1}\right)^{\prime }$ (*j* = 1, …, *n*_*i*_) and ${e}_{ij}^k$ (k = 1, …, (K-1)) is defined as ***=***I(*Y*_*ij*_ = *k*) − P(*Y*_*ij*_ = *k*). Therefore, E[***s***_***i***_] **=** ***σ***_***i***_**.** Fisher’s scoring algorithm is used to update both ***θ*** and *r* from m-th iteration to (m + 1)-th iteration, written as

$$ \left(\begin{array}{c}{\boldsymbol{\theta}}^{\left(m+1\right)}\\ {}{r}^{\left(m+1\right)}\end{array}\right)=\left(\begin{array}{c}{\boldsymbol{\theta}}^{(m)}\\ {}{r}^{(m)}\end{array}\right)+{\boldsymbol{U}}^{\ast}{\left({\boldsymbol{\theta}}^{(m)},{r}^{(m)}\right)}^{-1}{\sum}_{i=1}^N{\boldsymbol{U}}_{\boldsymbol{i}}\left({\boldsymbol{\theta}}^{(m)},{r}^{(m)}\right) $$

where

$$ {\left.{\boldsymbol{U}}^{\ast}\left({\boldsymbol{\theta}}^{(m)},{r}^{(m)}\right)=-E\left[D{\sum}_{i=1}^N{U}_i\left({\boldsymbol{\theta}}^{(m)},{r}^{(m)}\right)\right]=\sum \limits_{i=1}^N\left(\begin{array}{cc}{\boldsymbol{D}}_{\boldsymbol{i}}^{\prime }{\boldsymbol{V}}_{\boldsymbol{i}}^{-\mathbf{1}}{\boldsymbol{D}}_{\boldsymbol{i}}& \mathbf{0}\\ {}{\boldsymbol{F}}_{\boldsymbol{i}}^{\prime}\frac{\partial {\boldsymbol{\sigma}}_{\boldsymbol{i}}}{\partial {\boldsymbol{\theta}}^{\prime }}& {\boldsymbol{F}}_{\boldsymbol{i}}^{\prime}\frac{\partial {\boldsymbol{\sigma}}_{\boldsymbol{i}}}{\partial r}\end{array}\right)\right|}_{\boldsymbol{\theta} ={\boldsymbol{\theta}}^{(m)},\kern0.75em r={r}^{(m)},} $$

and *D* stands for the first-order derivative with respect to (***θ***, *r*), until the pre-specified convergence criterion is met. Estimates of multinomial logistic regression and r = 0 or 0.5 usually work well in terms of starting values.

Note the score equations will be reduced to the following GEE form [20] when applied to N unrelated samples. The coefficients estimation will follow the same iteratively reweighted least square method of generalized linear model [21] for multinomial outcome until a pre-specified convergence criterion is met.

$$ \boldsymbol{U}={\sum}_{i=1}^N{\boldsymbol{U}}_{\boldsymbol{i}}\left(\boldsymbol{\theta} \right)={\sum}_{i=1}^N{\boldsymbol{D}}_{\boldsymbol{i}}^{\prime }{\boldsymbol{V}}_{\boldsymbol{i}}^{-\mathbf{1}}\left({\boldsymbol{y}}_{\boldsymbol{i}}-{\boldsymbol{\mu}}_{\boldsymbol{i}}\right)=\mathbf{0} $$

### Robust score test

To determine if a genetic variant is associated with a multi-category phenotype, the following null hypothesis is tested *H*_0_ : ***β*** = **0**. We first define the score vectors $$ {\boldsymbol{U}}^{\left(\mathbf{1}\right)}=\left(\begin{array}{c}\begin{array}{c}{\boldsymbol{U}}_{{\boldsymbol{\upgamma}}_{\mathbf{1}}}\\ {}\boldsymbol{\vdots}\\ {}\boldsymbol{\vdots}\end{array}\\ {}{\boldsymbol{U}}_{{\boldsymbol{\upgamma}}_{\boldsymbol{K}-\mathbf{1}}}\end{array}\right) $$**,** $$ {\boldsymbol{U}}^{\left(\mathbf{2}\right)}={\boldsymbol{U}}_{\boldsymbol{\upbeta}}=\left(\begin{array}{c}{U}_{\upbeta_1}\\ {}\boldsymbol{\vdots}\\ {}{U}_{\upbeta_{K-1}}\end{array}\right) $$**,** *U*^(3)^ = *U*_*r*_. The score statistic is proposed as follows:

$$ \mathrm{s}={\left(\boldsymbol{A}\left({\hat{\boldsymbol{\theta}}}_{\mathbf{0}},{\hat{\boldsymbol{r}}}_{\mathbf{0}}\right){\boldsymbol{U}}^{\boldsymbol{main}}\left({\hat{\boldsymbol{\theta}}}_{\mathbf{0}},{\hat{\boldsymbol{r}}}_{\mathbf{0}}\right)\right)}^{\prime }{\left\{\boldsymbol{A}\left({\hat{\boldsymbol{\theta}}}_{\mathbf{0}},{\hat{\boldsymbol{r}}}_{\mathbf{0}}\right){\sum}_{i=1}^N\left[{\boldsymbol{U}}_{\boldsymbol{i}}^{\boldsymbol{main}}\left({\hat{\boldsymbol{\theta}}}_{\mathbf{0}},{\hat{\boldsymbol{r}}}_{\mathbf{0}}\right){\boldsymbol{U}}_{\boldsymbol{i}}^{\boldsymbol{main}}\left({\hat{\boldsymbol{\theta}}}_{\mathbf{0}},{\hat{\boldsymbol{r}}}_{\mathbf{0}}\right)\prime \right]\boldsymbol{A}\left({\hat{\boldsymbol{\theta}}}_{\mathbf{0}},{\hat{\boldsymbol{r}}}_{\mathbf{0}}\right)\prime \right\}}^{-1}\boldsymbol{A}\left({\hat{\boldsymbol{\theta}}}_{\mathbf{0}},{\hat{\boldsymbol{r}}}_{\mathbf{0}}\right){\boldsymbol{U}}^{\boldsymbol{main}}\left({\hat{\boldsymbol{\theta}}}_{\mathbf{0}},{\hat{\boldsymbol{r}}}_{\mathbf{0}}\right) $$

Where ${\hat{\boldsymbol{\theta}}}_{\mathbf{0}},{\hat{\boldsymbol{r}}}_{\mathbf{0}}$ are parameter estimates under *H*_0_ : ***β*** = **0.** $$ {\boldsymbol{U}}^{\boldsymbol{main}}\left(\boldsymbol{\theta}, r\right)=\left(\begin{array}{c}{\boldsymbol{U}}^{\left(\mathbf{1}\right)}\\ {}{\boldsymbol{U}}^{\left(\mathbf{2}\right)}\end{array}\right)={\sum}_{i=1}^N{\boldsymbol{U}}_{\boldsymbol{i}}^{\boldsymbol{main}}\left(\boldsymbol{\theta}, r\right) $$, ***A***(***θ***, *r*) = (−***U***^***∗***^_21_***U***^***∗***^_11_^−1^,  ***I***) with subscript 2 denoting rows/columns that correspond to **β,** subscript 1 denoting rows/columns that correspond to **γ**_**1**_***,*** …***,*γ**_***K −*** **1**_**,** and ***I*** is an identity matrix of size (K-1).

The score statistic follows a ${\chi}_{K-1}^2$ asymptotically according to the derivation for bivariate association testing in family samples [17, 22]. One of the major advantages is its robustness to incorrect variance specification. If the variance ***V***_***i***_ (i = 1, … N) is pre-specified correctly, then *var*(***U***^***main***^(***θ***, *r*)) will equal to ***U***^∗^ restricted to **β**, **γ**_**1**_***,*** …***,*γ**_***K −*** **1**_**,** and the score statistic will be simplified to

$$ \mathrm{s}={\left({\boldsymbol{U}}^{\left(\mathbf{2}\right)}\left({\hat{\boldsymbol{\theta}}}_{\mathbf{0}},{\hat{\boldsymbol{r}}}_{\mathbf{0}}\right)\right)}^T{\left\{{V}^{\left(\mathbf{2}\right)}\left({\hat{\boldsymbol{\theta}}}_{\mathbf{0}},{\hat{\boldsymbol{r}}}_{\mathbf{0}}\right)\right\}}^{-1}{\boldsymbol{U}}^{\left(\mathbf{2}\right)}\left({\hat{\boldsymbol{\theta}}}_{\mathbf{0}},{\hat{\boldsymbol{r}}}_{\mathbf{0}}\right). $$

where ${V}^{\left(\mathbf{2}\right)}={I}_{22}-{I}_{2\left(-2\right)}{I}_{\left(-2\right)\left(-2\right)}^{-1}{I}_{\left(-2\right)2}$ (The subscript 2 denotes the (K-1) row/columns corresponding to β_1_, …, β_(*K* − 1)_; “-“denotes excluding these rows/columns) and $I={\sum}_{\boldsymbol{i}=\mathbf{1}}^{\boldsymbol{N}}{\boldsymbol{D}}_{\boldsymbol{i}}^{\prime }{\boldsymbol{V}}_{\boldsymbol{i}}^{-\mathbf{1}}{\boldsymbol{D}}_{\boldsymbol{i}}$**.**

### Wald test

The Wald test is an alternative test with lower computational efficiency when applied to a large-scale genetic study. The Wald test statistic is proposed as follows:

$$ w=\left({\hat{\beta}}_1\kern0.5em \dots \kern0.5em {\hat{\beta}}_{\left(K-1\right)}\right)V{\left(\begin{array}{ccc}{\hat{\beta}}_1& \dots & {\hat{\beta}}_{\left(K-1\right)}\end{array}\right)}^{-1}\left(\begin{array}{ccc}{\hat{\beta}}_1& \dots & {\hat{\beta}}_{\left(K-1\right)}\end{array}\right)^{\prime } $$

This test statistic follows a ${\chi}_{K-1}^2$ asymptotically. The parameters $\hat{\boldsymbol{\theta}},\hat{r}$ are obtained from the score equations with no constraints (i.e. *H*_0_ ∪ *H*_*a*_) until the pre-specified convergence criterion is met.

The full variance matrix of all parameters $V\left(\hat{\boldsymbol{\theta}},\kern0.5em \hat{r}\ \right)$ is derived as $V\left(\hat{\boldsymbol{\theta}},\kern0.5em \hat{r}\ \right)={\boldsymbol{U}}^{\ast}{\left(\hat{\boldsymbol{\theta}},\hat{r}\right)}^{-1}{\sum}_{i=1}^N{\boldsymbol{U}}_{\boldsymbol{i}}\left(\hat{\boldsymbol{\theta}},\hat{r}\right){\boldsymbol{U}}_{\boldsymbol{i}}\left(\hat{\boldsymbol{\theta}},\hat{r}\right)^{\prime }{\left({\boldsymbol{U}}^{\ast}{\left(\hat{\boldsymbol{\theta}},\hat{r}\right)}^{\prime}\right)}^{-1}$. $V\left({\hat{\beta}}_1\kern0.5em \dots \kern0.5em {\hat{\beta}}_{\left(K-1\right)}\right)$ is extracted from $V\left(\hat{\boldsymbol{\theta}},\kern0.5em \hat{r}\ \right)$, a sandwich-type variance estimator [19], with rows and columns corresponding to $\left({\hat{\beta}}_1\kern0.5em \dots \kern0.5em {\hat{\beta}}_{\left(K-1\right)}\right)$.

### Ordinal traits

Under the same framework, using the statistical theory of ordinal regression, the above score and Wald tests can be easily extended to test the association of a genetic variant with an ordinal trait for a family-based design. More specifically, because P(*Y*_*ij*_ = *k*) can be derived from P(*Y*_*ij*_ = *k*) = P(*Y*_*ij*_ ≤ *k*) − P(*Y*_*ij*_ ≤ *k* − 1) using proportional cumulative logit models, then the same EGEE equations are used for parameter estimation. However, the dimensions of EGEE equations are reduced and mathematical formulas of the matrix elements are derived differently due to the use of proportional cumulative logit models. A computer implementation for both multinomial and ordinal phenotypes is provided in Additional File 1-2.

### Simulations

We conduct type-I error and power simulation studies to evaluate the validity of our score test in assessing the association between single-nucleotide variants **(**SNVs) with different minor allele frequencies (MAF) and a categorical trait with four categories (“multinomial” trait), and compare the score test to the Wald test and the multinomial logistic regression which does not account for related samples. We then conduct simulations to assess the power of all three approaches.

#### Type-I error

We compare the type-I error rate of the robust score test to the Wald test as well as multinomial logistic regression (without accounting for related samples) in both family-based and unrelated designs. We simulate a 4-category trait under the null hypothesis that there is no genetic association with the trait, i.e. *H*_0_ : *β*_1_ = … = *β*_3_ = 0. Eight SNV scenarios with MAF ranging from 0.01 to 0.3 are explored. For each SNV scenario and sample design, 500,000 replicates are simulated and the type-I error rate is defined as the proportion of simulations significant at the threshold of 0.01, 0.001, and 0.0001. For family-based samples, we also have conducted simulations to evaluate the type-I error of robust score and Wald test when applied to ordinal traits, based on 500, 000 replicates for each MAF scenario.

**Fig. 5:**
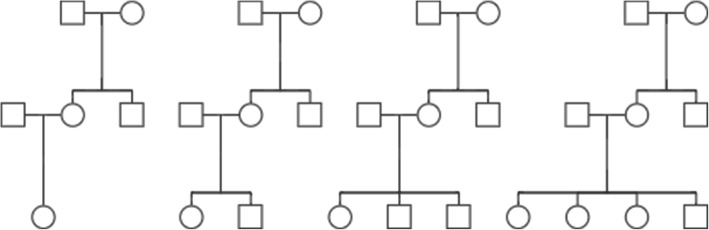
All possible family structures

*Family-based samples*: In each replicate, a total of 1000 independent 3-generation families with 2 grandparents who have one son and one daughter (Fig. 5**.**) are simulated. The number of grandchildren (3rd-generation) is randomly determined from a discrete uniform distribution ranging from 1 to 4. Within each of the 1000 families, we simulate additively coded genotypes (0, 1, or 2 minor alleles) of the grandparents under Hardy-Weinberg equilibrium, and the 2nd and 3rd generations’ genotypes are then simulated using random allele dropping. Two covariates (age and sex) are simulated. The sex of the 3rd-generation is randomly assigned, and the covariate of age is simulated in the following way [17]: we start by simulating the age of female offspring (2nd generation) from a continuous uniform distribution ranging from 25 to 50. Her spouse’s age is set to be within 5-year of her age. The male offspring’s ages (2nd generation) are set to be within 5 years of the sister with at least a 1-year gap to exclude twins. Then we simulate the age of the grandparents (1st generation). The grandmother is assumed to be 20 to 45 years older than both offspring (2nd generation), and the grandfather’s age is set to be within 5-year of the grandmother’s age and he must be at least 20 years older than his older offspring. Finally, we simulate the age of the 3rd generation, in such a way that everyone in the 3rd generation is assumed to be 20 to 45 years younger than the mother (2nd generation) and at least 20 years younger than the father (2nd generation). Two continuous traits are simulated from age and sex, based on the following two equations, i.e. age and sex explains around 3 and 0.002% of the total variance of the latent variable *u*_1_ versus 0.8 and 0.01% of the latent variable *u*_2_:

$$ {u}_1=5.6+0.025 age+0.5 sex+{\varepsilon}_1; $$

$$ {u}_2=30+0.04 age+0.2 sex+{\varepsilon}_2; $$

where $$ \left(\begin{array}{c}{\varepsilon}_1\\ {}{\varepsilon}_2\end{array}\right)\sim N\left(0,{\boldsymbol{\Sigma}}_{\boldsymbol{a}}\bigotimes \boldsymbol{\Phi} +{\boldsymbol{\Sigma}}_{\boldsymbol{e}}\bigotimes \mathbf{I}\right) $$, the additive covariance matrix is $$ {\boldsymbol{\Sigma}}_{\boldsymbol{a}}=\left(\begin{array}{cc}4& 6\\ {}6& 36\end{array}\right) $$ and the environmental covariance matrix is $$ {\boldsymbol{\Sigma}}_{\boldsymbol{e}}=\left(\begin{array}{cc}4& 6\\ {}6& 36\end{array}\right) $$. **Φ** is the relationship matrix which is a kinship matrix multiplied by 2.

We transform *u*_1_, *u*_2_ to two binary traits using a threshold model with a disease prevalence of 10 and 35%, assuming a disease with a moderate prevalence such as type 2 diabetes (T2D) and a high prevalence such as obesity. The multinomial trait is then defined by these two binary traits as follows: diabetes & obesity, diabetes but no obesity, obesity but no diabetes and no diabetes & no obesity, in adults.

*Unrelated samples:* In each replicate, we simulate a total of 5000 independent subjects with ages ranging from 18 to 90. A total of 5000 independent additively-coded genotypes are simulated. The sex is randomly assigned (1 = male; 2 = female). We then simulate two continuous traits influenced by age and sex only, based on the following two equations, so that age and sex explain around 3.2 and 0.8% of the total variance of *u*_1_ versus 0.94 and 0.01% of *u*_2_ respectively:

$$ {u}_1=5.6+0.025 age+0.5 sex+{\varepsilon}_1; $$

$$ {u}_2=30+0.04 age+0.2 sex+{\varepsilon}_2; $$

where $$ \left(\begin{array}{c}{\varepsilon}_1\\ {}{\varepsilon}_2\end{array}\right)\sim N\left(0,{\boldsymbol{\Sigma}}_{\boldsymbol{T}}\bigotimes \mathbf{I}\right) $$ with $$ {\boldsymbol{\Sigma}}_{\boldsymbol{T}}=\left(\begin{array}{cc}8& 12\\ {}12& 72\end{array}\right) $$. We transform *u*_1_, *u*_2_ as described in the family design section.

We evaluate the type-I error of the proposed score test and Wald test, and then compare them to the multinomial logistic regression assuming independence among observations (using likelihood ratio test (LRT))**.**

#### Power evaluation

We compare the power of the score to the Wald test and multinomial logistic regression under the same allele scenarios and with the same family/unrelated structure as described above. In addition to the effects of age and sex, we also include an additively coded genetic variant g which explains approximately 0.5% of the variance of each continuous trait, i.e.

$$ {u}_1=5.6+0.025 age+0.5 sex+\sqrt{\frac{4\times 0.01}{2 MAF\left(1- MAF\right)}}g+{\varepsilon}_1; $$

$$ {u}_2=30+0.04 age+0.2 sex+\sqrt{\frac{36\times 0.01}{2 MAF\left(1- MAF\right)}}g+{\varepsilon}_2; $$

With this phenotype generation model, both traits are simulated under the alternative hypothesis that there is an association between the trait and the genetic variant. For each MAF scenario, a total of 5000 replicates are generated. The power rate is then evaluated for 3 different significance thresholds including the commonly used GWAS threshold for each method.

### Framingham heart study

The motivation for developing this efficient score test is to make the application to a large-scale genetic study computationally feasible, especially after the cost of whole-genome sequencing has been greatly reduced in recent years.

We apply the robust score test to the Framingham Heart Study (FHS) [17, 23]. A total of 7564 participants from 1315 families are analyzed, after excluding observations with missing values in body mass index (BMI), age, sex, the first 10 principal components (PC) s or T2D status. The primary outcome is diabesity with four categories as defined above. Diabesity is considered a modern epidemic and the largest in human history [24]. However, there are very few papers available regarding genetic association studies on this trait. We analyze the association between diabesity and genotypes from the Framingham SNP Health Association Resource (SHARe) project sponsored by the National Heart, Lung and Blood Institute (NHLBI), adjusting for age, sex, and the first 10 PCs. Genotypes from Affymetrix 550 K genotyping arrays (Affymetrix, Santa Clara, CA, USA), supplemented by the Affymetrix MIPS array, are available on 8481 participants after exclusion for low call rate (< 97%), heterozygosity rate outside of 5 SDs from the mean or excess Mendelian errors (> 1000). Additional SNVs are imputed with the software MACH (Markov Chain-based haplotyper) using the HapMap 2 reference haplotypes [25]. To help understand the GWAS results of diabesity and given the fact that diabesity is jointly constructed from obesity and diabetes, we perform two additional family-based logistic regression analyses using our approach to study the association of diabetes and genotypes, and the association of obesity and genotypes respectively. A secondary outcome treats the diabesity as an ordinal variable with 4 levels of increasing severity. We apply both our ordinal approach and POLMM with derived sparse GRM matrix to the secondary outcome and compare the results.

## Supplementary Information

**Additional file 1.**

**Additional file 2.**

**Additional file 3.**

## Data Availability

The FHS dataset that supports the findings of this manuscript is available on dbGap (dbGaP Study Accession: phs000007.v32.p13, https://www.ncbi.nlm.nih.gov/projects/gap/cgi-bin/study.cgi?study_id=phs000007.v32.p13).

## Abbreviations

GLMM: Generalized linear mixed model
EGEE: Extended generalized estimating equations
SNV: Single-nucleotide variant
MAF: Minor allele frequency
T2D: Type-2 diabetes
FHS: Framingham heart study
BMI: Body mass index
SHARe: SNP Health Association Resource
NHLBI: National Heart, Lung and Blood Institute
MACH: Markov Chain-based haplotyper
POLMM: Proportional Odds Logistic Mixed Model
