# Supplementary material for: Genetic association tests in family samples for multi-category phenotypes
Source: BMC Genomics. 2021 Dec 4;22:873. doi: 10.1186/s12864-021-08107-x (PMC8642939; doi:10.1186/s12864-021-08107-x)
Supplement: Supplementary file 3 — Additional file 3. [file 12864_2021_8107_MOESM3_ESM.docx]

| 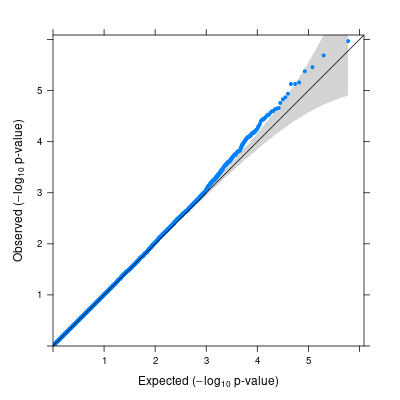   1. Multinomial logit using robust score test: lambda=1.01 | 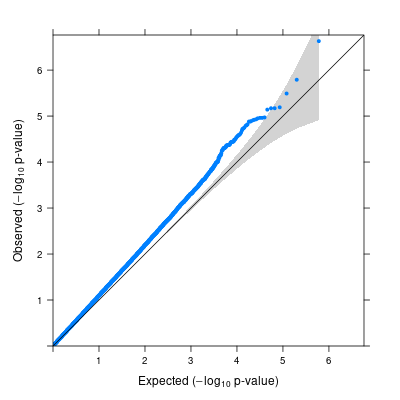   1. Multinomial logit using simplified score test: lambda=1.17 |
| --- | --- |
| 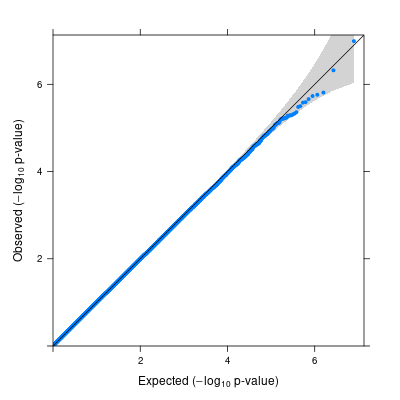   1. Cumulative logit using robust score test: lambda=1.007 | 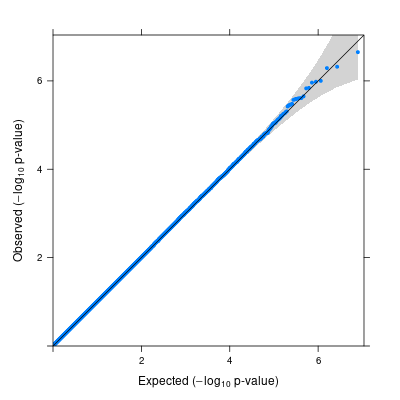   1. Cumulative logit using simplified score test: lambda=1.009 |

SFigure 1. Plot (a), (c) are based on all p-values from all MAF scenarios in the column of “robust score test” in table 1, 3; plot (b) and (d) are based on all p-values from all MAF scenarios using simplified score test statistics under the section “**Robust Score Test**”.

**
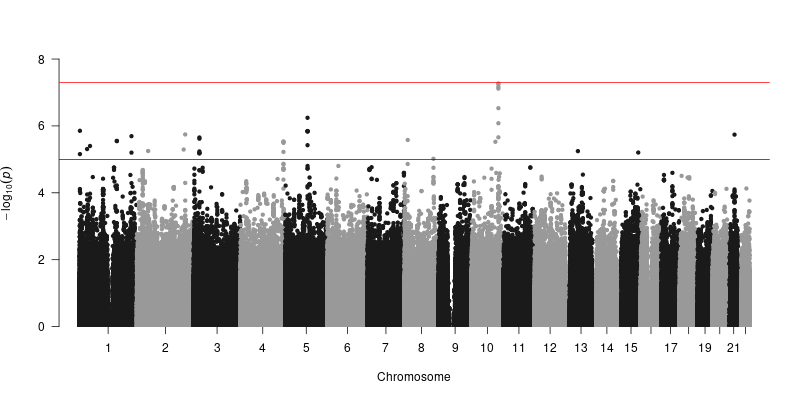
**

SFigure 2. Manhattan plot of diabetes


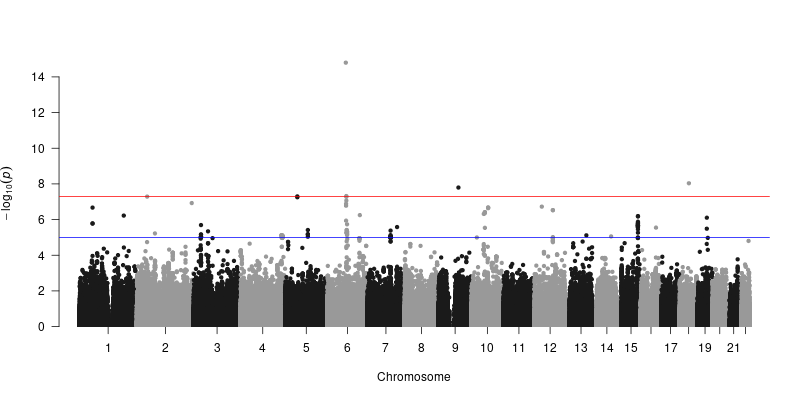


SFigure 3. Manhattan plot of obesity
